# Percutaneous Revascularization for Ischemic Left Ventricular Dysfunction: Cost-Effectiveness Analysis of the REVIVED-BCIS2 Trial

**DOI:** 10.1161/CIRCOUTCOMES.123.010533

**Published:** 2023-11-06

**Authors:** Carlos Chivardi, Holly Morgan, Mark J. Sculpher, Tim Clayton, Richard Evans, Matthew Dodd, Mark Petrie, Christopher A. Rinaldi, Peter O'Kane, Louise Brown, Divaka Perera, Pedro Saramago

**Affiliations:** Centre for Health Economics, University of York, United Kingdom (C.C., M.J.S., P.S.).; British Heart Foundation Center of Research Excellence, School of Cardiovascular Medicine and Sciences, King’s College London, United Kingdom (H.M., D.P.).; Clinical Trials Unit, London School of Hygiene and Tropical Medicine, United Kingdom (T.C., R.E., M.D.).; Cardiology Department, Institute of Cardiovascular and Metabolic Sciences, University of Glasgow, United Kingdom (M.P.).; Cardiology Department, Guy’s and St Thomas’ Hospital NHS Foundation Trust, London, United Kingdom (A.R., D.P.).; Cardiology Department, Royal Bournemouth and Christchurch Hospital, Bournemouth, United Kingdom (P.O.).; MRC Clinical Trials Unit, University College London, United Kingdom (L.B.).; (principal investigator); (coinvestigators); (coordinators); (principal Investigator); (Coinvestigators); (Coordinators); (principal investigator); (coinvestigators); (coordinators); (principal investigator); (coinvestigator); (coordinators); (principal investigator); (coinvestigators); (coordinators); (principal investigator); (coinvestigators); (coordinators); (principal investigator); (coinvestigators); (coordinators); (principal investigator); (coinvestigators); (coordinators); (principal investigator); (coinvestigators); (coordinators); (principal investigator); (coinvestigators); (coordinators); (principal investigator); (coinvestigator); (coordinators); (principal investigator); (coinvestigator); (coordinators); (principal investigator); (coinvestigator); (coordinators); (principal investigator); (coinvestigators); (coordinators); (principal investigator); (coinvestigators); (coordinators); (principal investigator); (coinvestigator); (coordinator). Sunderland Royal Hospital; (principal investigator); (coinvestigator); (coordinators); (principal investigator); (coinvestigators); (coordinators); (principal investigator); (coinvestigators); (coordinators); (principal investigator); (coinvestigators); (coordinators); (principal investigator); (coinvestigator); (coordinator). University Hospitals Coventry and Warwickshire; (principal investigator); (coinvestigator); (coordinators); (principal investigator); (coinvestigator); (coordinator); (principal investigator); (coinvestigators); (coordinators); (principal investigator); (coordinator). Worcestershire Acute Hospitals; (principal investigator); (coinvestigator); (coordinator). Worthing Hospital; (principal investigator); (coinvestigator); (coordinator). Blackpool Victoria Hospital; (principal investigator); (coinvestigator); (coordinator). Dorset County Hospital; (principal investigator); (coinvestigators); (coordinators); (principal investigator); (coinvestigator); (coordinator). Birmingham Heartlands Hospital; (principal investigator); (coinvestigators); (coordinator). Great Western Hospital, Swindon; (principal investigator); (coordinator). Ninewells Hospital, Dundee; (principal investigator); (coinvestigators); (coordinator). Northern General Hospital, Sheffield; (principal investigator); (coinvestigator); (coordinator). Queen Alexandra Hospital, Portsmouth; (principal investigator); (coinvestigator); (coordinator). Royal Oldham Hospital; (principal investigator); (coinvestigator); (coordinator). Basingstoke and North Hampshire Hospital; (principal investigator); (coinvestigator); (coordinator). North Wales Cardiac Centre; (principal investigator); (coinvestigator); (coordinator). The York Hospital; (principal investigator); (coinvestigator); (coordinators)

**Keywords:** coronary artery disease, heart failure, humans, myocardial revascularization, percutaneous coronary intervention

## Abstract

**BACKGROUND:**

Percutaneous coronary intervention (PCI) is frequently undertaken in patients with ischemic left ventricular systolic dysfunction. The REVIVED (Revascularization for Ischemic Ventricular Dysfunction)-BCIS2 (British Cardiovascular Society-2) trial concluded that PCI did not reduce the incidence of all-cause death or heart failure hospitalization; however, patients assigned to PCI reported better initial health-related quality of life than those assigned to optimal medical therapy (OMT) alone. The aim of this study was to assess the cost-effectiveness of PCI+OMT compared with OMT alone.

**METHODS:**

REVIVED-BCIS2 was a prospective, multicenter UK trial, which randomized patients with severe ischemic left ventricular systolic dysfunction to either PCI+OMT or OMT alone. Health care resource use (including planned and unplanned revascularizations, medication, device implantation, and heart failure hospitalizations) and health outcomes data (EuroQol 5-dimension 5-level questionnaire) on each patient were collected at baseline and up to 8 years post-randomization. Resource use was costed using publicly available national unit costs. Within the trial, mean total costs and quality-adjusted life-years (QALYs) were estimated from the perspective of the UK health system. Cost-effectiveness was evaluated using estimated mean costs and QALYs in both groups. Regression analysis was used to adjust for clinically relevant predictors.

**RESULTS:**

Between 2013 and 2020, 700 patients were recruited (mean age: PCI+OMT=70 years, OMT=68 years; male (%): PCI+OMT=87, OMT=88); median follow-up was 3.4 years. Over all follow-ups, patients undergoing PCI yielded similar health benefits at higher costs compared with OMT alone (PCI+OMT: 4.14 QALYs, £22 352; OMT alone: 4.16 QALYs, £15 569; difference: −0.015, £6782). For both groups, most health resource consumption occurred in the first 2 years post-randomization. Probabilistic results showed that the probability of PCI being cost-effective was 0.

**CONCLUSIONS:**

A minimal difference in total QALYs was identified between arms, and PCI+OMT was not cost-effective compared with OMT, given its additional cost. A strategy of routine PCI to treat ischemic left ventricular systolic dysfunction does not seem to be a justifiable use of health care resources in the United Kingdom.

**REGISTRATION:**

URL: https://www.clinicaltrials.gov; Unique identifier: NCT01920048.

**WHAT IS KNOWN**

- Percutaneous coronary intervention (PCI) is frequently utilized in patients with ischemic left ventricular systolic dysfunction.
- The REVIVED (Revascularization for Ischemic Ventricular Dysfunction)-BCIS2 (British Cardiovascular Society-2)trial demonstrated that PCI did not decrease all-cause death or heart failure hospitalization rates although patients undergoing PCI initially reported an improved health-related quality of life compared with optimal medical therapy alone.
- The economic and health consequences of a PCI strategy for ischemic left ventricular systolic dysfunction remain unknown.

**WHAT THE STUDY ADDS**

- This study evaluates the economic implications associated with PCI in ischemic left ventricular systolic dysfunction patients, filling a crucial knowledge gap.
- Results indicate that although PCI provides similar health benefits, it comes at a higher cost when compared with optimal medical therapy alone.
- Most health resource utilization occurred in the first 6 months post-randomization, related to the PCI procedures.
- Study findings indicate that routine PCI as a treatment strategy for ischemic left ventricular systolic dysfunction is not cost-effective, which has implications for health care resource allocation.

**See Editorial by Girotra and Kumbhani**

Heart failure (HF) is an increasing worldwide health problem, with coronary artery disease being the most common cause.^1^ Over the last few decades, advances in medical and device therapy have been central to improving the prognosis of patients with ischemic HF. Coronary revascularization is frequently used as an adjunct to medical therapy (MT) in these patients, with the benefits of coronary artery bypass grafting (CABG) and percutaneous coronary intervention (PCI) having been evaluated in the STICH trial (Surgical Treatment for Ischemic Heart Failure) and the REVIVED trial (Revascularization for Ischemic Ventricular Dysfunction), respectively.^2,3^

In the REVIVED trial, it was hypothesized that revascularization with PCI in addition to optimal MT (OMT) compared with OMT alone could improve event-free survival in patients with severe ischemic left ventricular systolic dysfunction (ILVD).^4^ There was no difference between groups in the occurrence of the primary outcome at a median of 3.4 years. Early health-related quality of life was better in patients assigned to have PCI, and although this difference was not sustained, this has led some to conclude that PCI is still a beneficial strategy.^5^

The aim of our current analysis was to assess the cost-effectiveness of PCI for patients with severe stable ILVD by using patient-level resource use and patient-reported outcome data collected in the REVIVED trial.

## METHODS

### Trial Design and Patient Population

REVIVED was a prospective, multicenter, randomized, open-label trial involving 700 patients with ILVD^3^; randomization was to either PCI+OMT or OMT alone. Participants were enrolled between 2013 and 2020; full patient eligibility criteria have been published previously.^4^ The trial protocol was registered before the enrollment of the first patient (https://www.clinicaltrials.gov; NCT01920048) and was approved by the UK Health Research Authority. All patients provided written informed consent. The data that support the findings of this study and the analytic methods will be made available 1 year from the completion of the trial on reasonable request to the corresponding author.

The patients enrolled had a median age of 69 years, 12% were female, the median British Cardiovascular Intervention Society jeopardy score was 10, the mean baseline LVEF was 27%±6.8%, and 26% had New York Heart Association class III or IV functional status (Table S1). The baseline Kansas City Cardiomyopathy Questionnaire (KCCQ) mean overall score was 60.9, and the EuroQol 5-dimension 5-level (EQ-5D-5L) mean utility score was 0.67.

### Economic Analysis

The analysis follows the preferred methods of the UK National Institute for Health and Care Excellence^6^ and takes a UK National Health Service (NHS) and Personal Social Services perspective. A time horizon of up to 8 years was used based on trial follow-up, with costs and consequences discounted at a 3.5% annual rate.^6^ Economic evaluation results were expressed using differences in costs and quality-adjusted life-years (QALYs) between the treatment options.

### Estimation of Costs

Health care resources used by each participant in the trial were obtained from the trial case report forms completed by trial investigators. The case report form captured the health resource consumption at baseline, 6 months, 12 months, and then on a yearly basis, with only postrandomization consumption included for analysis. Relevant health resources included revascularization, prescribed medications, HF hospital admissions and related treatments, implantable cardiac device implantation or upgrade, outpatient visits, and clinical investigations (eg, echocardiogram) during the first 2 years following randomization. HF hospitalizations, unplanned revascularizations, and implantable device information were collected yearly for 2 to 8 years. Unit costs for the health resources were obtained from National NHS Reference Costs databases^7^ (Table S2), and medication prices were obtained from the British National Formulary.^8^

Health care resource use was combined with relevant unit costs and aggregated to produce a total cost for each trial participant. Six distinct cost categories were defined: planned and unplanned revascularization procedures, medications, hospitalization, implanted devices, and clinical investigations (including hemoglobin, creatinine, cholesterol, low density lipoprotein, high density lipoprotein, triglyceride, B-type natriuretic peptide, hemoglobin A1c, and echocardiogram). Within the intervention arm, planned revascularization procedures encompassed the costs associated with the initial planned percutaneous coronary procedures and their subsequent stages. Unplanned revascularization is related to any subsequent unplanned PCIs or CABG procedures throughout trial follow-up. Medication costs are related to cardiac medication at randomization, at hospital discharge, and relevant assessment points of follow-up in both arms of the trial. The cost of medication was estimated according to the dosage and duration of each drug consumed. HF hospitalization costs include costs related to inpatient stays, as well as any associated diagnostic tests, procedures, and medication required within that admission. Implantable device costs encompassed the device itself (cardiac resynchronization therapy, cardiac resynchronization therapy with defibrillator, or implantable cardioverter defibrillator only) and all costs associated with its implantation or upgrade. Finally, clinical investigation costs were related to the diagnosis and management of coronary artery disease and HF, including costs associated with tests such as blood tests and echocardiograms (prerandomization eligibility testing was not considered).

By estimating the costs associated with each of these categories, we aimed to provide a comprehensive understanding of the cost burden associated with treating patients with ILVD. Costs were expressed as total per-participant costs over the follow-up period.

### Health-Related Quality of Life

In the REVIVED trial, health-related quality of life was assessed using the KCCQ and the EQ-5D-5L questionnaires.^9^ Health-related quality-of-life data were collected at baseline, 6 months, 12 months, and annually thereafter until the trial ended. The current analysis used the EQ-5D-5L, a standardized instrument for measuring health-related quality of life that consists of 5 domains: mobility, self-care, usual activities, pain/discomfort, and anxiety/depression. Each domain is scored on a 5-point scale (no problems, slight problems, moderate problems, severe problems, and unable), and the response of each trial participant is converted into a single “utility” score, ranging from 0 (representing death) to 1 (representing perfect health). Utilities are based on the preferences of a sample of the UK population.^10,11^ For each individual participant, QALYs were derived from the utilities for each year of follow-up, considering zero QALYs for deceased patients.

### Multiple Imputation

Missing responses to the EuroQol 5-dimension (EQ-5D) instrument were imputed using multiple imputations by chained equations.^12^ Two types of missing values were imputed: (1) missing values resulting from questionnaires that were not completed during follow-up were and intended to have been collected via the case report form and (2) values beyond the last date of follow-up, up to a hypothetical follow-up duration of 8 years, where the actual patient follow-up duration was shorter. The overall missing rate in the OMT group was 5.0% and 4.6% for the PCI group. Results from imputed data sets were combined using Rubin rules to obtain joint regression model estimates.^12^ Further detail on the multiple imputation approach can be found in the Supplemental Material and Table S3.

### Regression Analysis

Generalized linear models were used to estimate independently predicted total costs and QALYs over the follow-up period. Adjustment for clinically relevant and validated covariates was performed, consistent with the primary outcome analysis.^4,12^ Covariable adjustment included the following baseline characteristics: age centered (continuous), sex (binary: F/M), New York Heart Association class (categorical: I [reference], II, III, and IV), BMI (continuous), ethnicity (categorical: White [reference], Asian, Afro-Caribbean, and other), British Cardiovascular Intervention Society jeopardy score (categorical: mild, 2–4; moderate, 6–8; and severe, 10–12), smoking status (categorical: never [reference], current, and ex-smoker), previous HF hospitalization (binary: Y/N), previous PCI and/or CABG (binary: Y/N), previous myocardial infarction (binary: Y/N), hypertension (binary: Y/N), and diabetes (binary: Y/N). The modeling of total QALYs also considered patients’ baseline EQ-5D utilities (continuous) as a covariable. Different distributional assumptions (log-normal, gen gamma, and Gaussian) and link functions (identity and log) were tested for the total costs and QALY models. The model selection process was based on the distributional properties of the dependent variables, their statistical fit as assessed by Akaike information criteria/Bayesian information criteria statistics,^13^ and notably, considering the suitability and reasonableness of the estimated or predicted outcomes generated by the selected statistical model.

To consider the potential interdependence and correlation between costs and outcomes, a seemingly unrelated regression model was performed as a modeling alternative.^14^ Seemingly unrelated regression is considered particularly useful when analyzing complex systems or data sets where multiple dependent variables, such as costs and QALYs here, are expected to have shared underlying factors.^14^

### Cost-Effectiveness Analysis

Incremental cost-effectiveness analysis was conducted based on differences in mean costs and QALYs between the 2 randomized groups. In the context of 1 group having higher mean costs and QALYs, incremental cost-effectiveness was estimated as the relevant intervention’s incremental cost per additional QALY. Judgments on the cost-effectiveness of interventions were performed by comparing the incremental cost-effectiveness ratio to the National Institute for Health and Care Excellence cost-effectiveness threshold range of £20 000 to £30 000 per QALY gained.

Sensitivity analyses were performed to assess the robustness of the results. The analyses were conducted to evaluate the impact of key assumptions and uncertainties on the estimated incremental cost-effectiveness ratio and to test the validity of the findings. A probabilistic sensitivity analysis was considered to account for the joint uncertainty in all parameters simultaneously based on 1000 random samples from the parameter distributions, enabling the estimation of the probability of each intervention being cost-effective at a range of cost-effectiveness thresholds. A scenario analysis was also considered by varying the unit cost of implantable devices (CRT, CRT-D, and ICD only), covering values reported in 2 different sources (Table S2).

## RESULTS

Between 2013 and 2020, 347 patients were randomized to PCI+OMT and 353 to OMT alone. A total of 225 patients (32%) died during the median trial follow-up of 41 months. The KCCQ overall summary score at 6 months increased by 6.5 points more in the PCI+OMT arm (+11.2 versus +4.7). However, by 24 months, the difference was not significant (70.6 versus 68.1, a difference of 2.6); the same trend was also seen in EQ-5D-5L.^3^ By the end of trial follow-up, 53.1% of patients had a cardiac device in situ, with 93 patients in the PCI+OMT and 120 patients in the OMT arm having a device inserted or upgraded after randomization.^15^ About unplanned revascularizations, there were 10 (2.9%) and 37 (10.5%) procedures in the PCI+OMT and OMT arms, respectively.

### Resource Use and Costs

**Figure 1.**
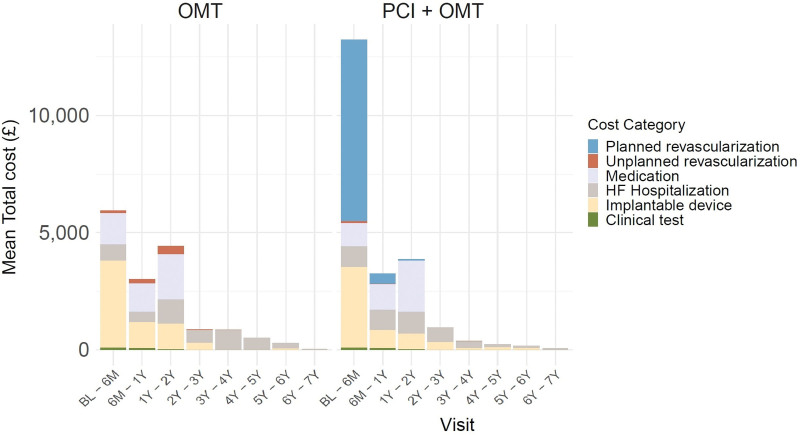
**Breakdown mean total costs by treatment group over all years.** Planned revascularizations, medication, and clinical test information were not collected from 2years onward. BL indicates baseline; HF, heart failure; OMT, optimal medical therapy; and PCI, percutaneous coronary intervention.

In the OMT group, the unadjusted mean total cost per patient during all follow-ups was £15 882 (95% CI, £13 958–£17 806), while for the PCI+OMT group, it was £21 674 (95% CI, £19 722–£23 626). The mean difference between the groups was £5791 (95% CI, £3056–£8528; Table 1). Figure 1 displays the distribution of unadjusted mean costs by category over the follow-up period for each treatment group. The higher unadjusted mean costs for PCI+OMT were driven by the assigned treatment (planned revascularization mean cost: £7752) performed during the first 6 months post-randomization (n=325 [94%], patients received a planned PCI). A total of 417 PCI procedures were performed on 334 patients; 80 patients had at least 1 staged procedure (Table 1). All other costs were broadly similar between the groups across the whole duration of follow-up, with implantable devices, medication, and HF-related hospitalizations having a substantial impact on total costs in both groups. More patients in the OMT group received implantable devices following randomization than the PCI+OMT group although this difference was not statistically significant (34% versus 27%). Although the frequency of hospitalizations for HF was similar (PCI+OMT: n=103, 29%; OMT alone: n=108, 31%), patients hospitalized in the OMT group spent on average more days in hospital (mean, 3.14 [95% CI, –2.88 to 9.49] days) due to HF than the PCI+OMT group.

**Table 1.**
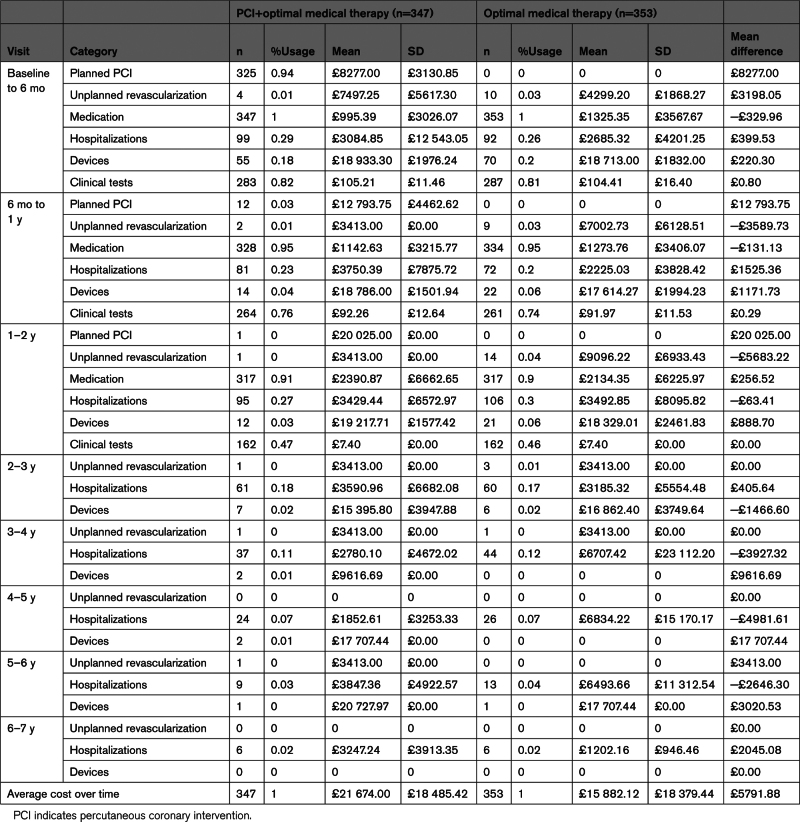
Breakdown of Total Cost per Visit

Most of the within-trial costs were incurred during the first 2 years of randomization in both groups. The average cost per patient during the first 2 years was £13 366 for the OMT group and £19 905 for the PCI+OMT group. Apart from the cost of the randomized intervention, the 2 groups had similar resource consumption (equivalent medication and clinical investigation usage up to 2 years) and costs (Tables S4 through S7).

### Health-Related Quality of Life

**Figure 2.**
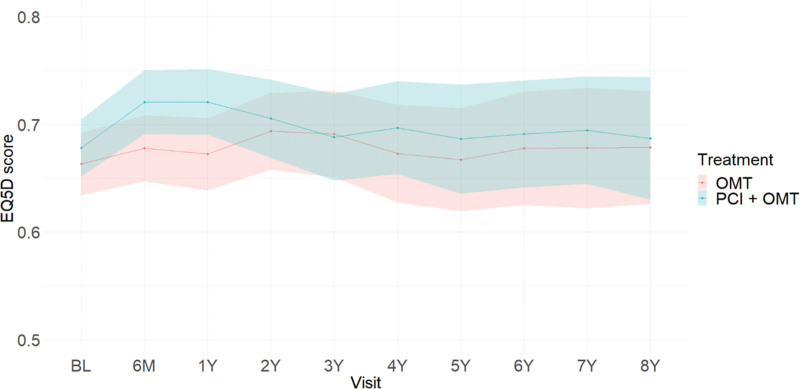
**Postimputation EuroQol 5-dimension (EQ-5D) score time trend by treatment group across follow-up (excluding those who have died).** Shaded areas represent 95% CIs. BL indicates baseline; OMT, optimal medical therapy; and PCI, percutaneous coronary intervention.

Table 2 presents observed EQ-5D-5L utilities for the 2 treatment groups, PCI and OMT, across the follow-up. A higher mean utility for PCI+OMT than for OMT was observed up to 1 year with minimal difference thereafter. Over the course of the study, the observed mean utility for PCI+OMT was the same or higher than for OMT. Figure 2 provides a time trend of imputed EQ-5D index scores by treatment group, which reinforces these findings. Patients in the PCI+OMT group accrued, on average, 0.527 unadjusted EQ-5D score, while patients randomized to the OMT alone group gained on average 0.509 unadjusted EQ-5D score. Table S8 presents the observed total QALYs.

**Table 2.**
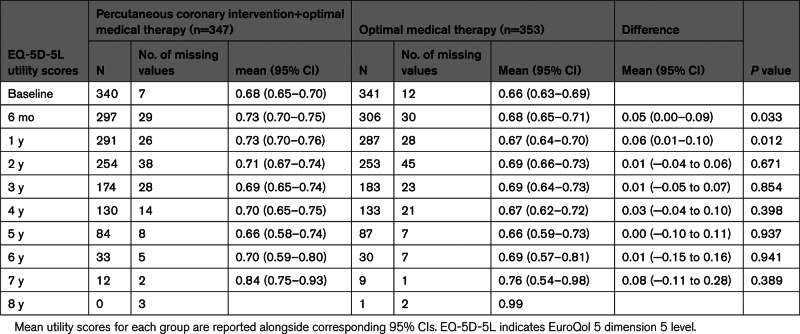
Observed EuroQol 5-Dimension Index Scores

### Regression Analysis

Table S9 highlights the baseline characteristics that were identified to have influenced total costs and QALYs. The randomized treatment group was important in explaining the variation in total costs but not in total QALYs, with PCI+OMT associated with a higher cost (Figure 1). Findings suggested that older age was associated with lower QALYs (*P*<0.05), though having a nonsignificant impact on costs (*P*=0.27). BMI was found to be relevant to explain variation in total costs but not total QALYs. A comparison of the baseline characteristics of patients with higher predicted costs (≥£20 000) with the overall sample revealed that patients with higher consumption of health resources have a higher prevalence of diabetes (76% versus 41%) and previous PCI (35% versus 20%) and a greater proportion of class 3/4 New York Heart Association classification (53% versus 26%).

### Cost-Effectiveness

The predicted average cost for OMT alone, adjusted for differences in baseline covariables, was £15 569 (95% CI, £15 302–£15 835) compared with £22 352 (95% CI, £21 969–£22 734) for a strategy of PCI+OMT (Table 3). The predicted mean cost difference between the 2 strategies was £6782 (95% CI, £6666–£6899), indicating a substantial cost difference between strategies, in favor of OMT alone. The OMT group accrued 4.16 (95% CI, 4.02–4.30) QALYs over the follow-up period, adjusted for differences in baseline covariables, compared with 4.14 (95% CI, 4.02–4.27) in the PCI+OMT group, which results in an incremental predicted QALY difference of –0.015 (95% CI, –0.385 to 0.355) for a strategy of PCI+OMT. Thus, PCI+OMT is estimated to have a higher mean cost with lower mean QALYs compared with OMT alone, which means that it is dominated by OMT alone and not cost-effective. Table S8 presents the observed incremental costs and QALYs, which are aligned with the adjusted outcomes.

**Table 3.**
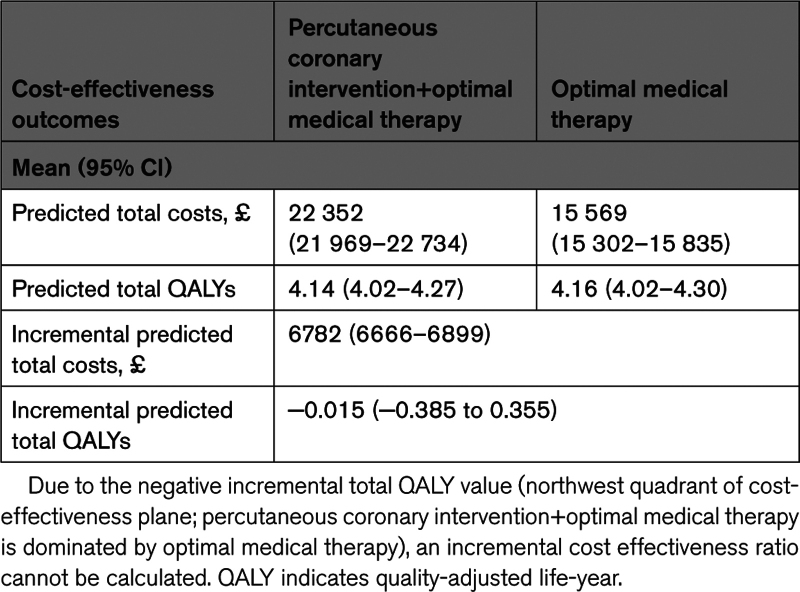
Cost-Effectiveness Results

**Figure 3.**
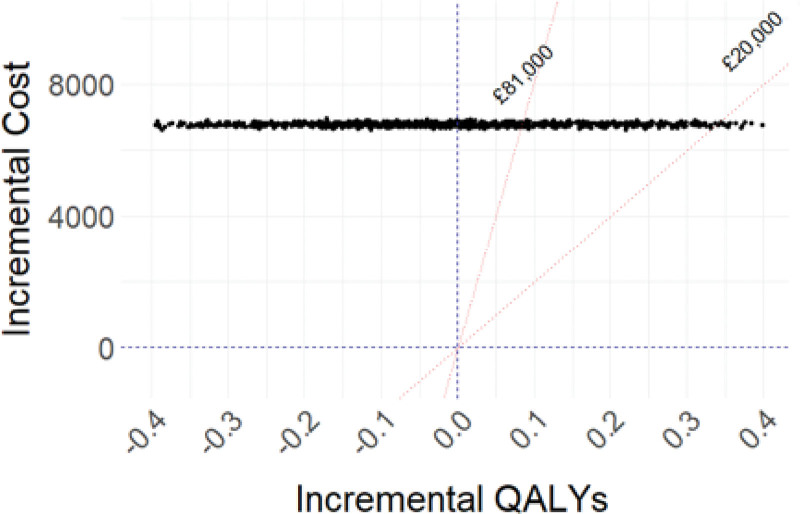
**Cost-effectiveness plane with probabilistic sensitivity analysis results.** £20 000 is the UK threshold and £82 000 is the US threshold using the current official exchange rate of £0.81. QALY indicates quality-adjusted life-year.

The probabilistic sensitivity analysis revealed that none of the simulations implemented resulted in a different outcome from the deterministic one (Figure 3). That is, even when accounting for parameter uncertainty, OMT alone continues to be less costly with slightly higher QALYs compared with PCI+OMT. Thus, the probability of PCI+OMT being cost-effective at the National Institute for Health and Care Excellence cost-effectiveness threshold (£20k to –£30k per QALY gained) was 0. Results of the seemingly unrelated regression model were found to be consistent with the outcomes of the main regression analysis, as no significant difference was identified between estimated QALYs for the control and treatment groups (Table S10). The scenario analysis examined the potential impact of changes in implant costs on treatment expenses and found that such variations did not result in significant cost fluctuations.

## DISCUSSION

In this prospective within-trial cost-effectiveness analysis of the REVIVED trial, no appreciable difference in total QALYs was identified between arms, but the PCI+OMT strategy was substantially more expensive than OMT alone, largely due to the upfront cost of PCI. Consequently, PCI+OMT was economically dominated and not cost-effective compared with OMT. When parameter uncertainty is allowed, there was no probability of PCI+OMT being cost-effective at a threshold of ≥£20 000 per QALY. The clinical results of REVIVED were neutral for the comparison of strategies, with no safety concerns raised with PCI, in contrast to the STICH trial where CABG was associated with a 3-fold to 4-fold excess in mortality within the first 2 years following randomization.^2^ Hence, the REVIVED clinical result for PCI has been interpreted as showing neither a clinical benefit nor any clinical downside to performing PCI in these patients. In REVIVED, the short-term impact of PCI on health-related quality of life captured in the KCCQ (overall score) and EQ-5D-5L (utility score) suggested a possible benefit relative to OMT and justified this economic evaluation. However, our economic findings are notable in showing that routine performance of PCI in these patients has a negative economic impact on health care systems. Given the high prevalence of patients with HF with severe ILVD and the expectation of an increased incidence over the medium to long term in the context of an aging population, substantial health care system savings are anticipated.

No prior study has reported on the incremental costs of the PCI against relevant comparators for patients with ILVD. Our findings are in contrast to the economic analysis performed on the STICH trial data.^2^ The STICH trial compared CABG+MT to MT alone in patients with ILVD and showed no overall difference in survival at 5 years although improved survival was demonstrated in the CABG arm at 10 years of follow-up.^16^ An economic analysis of the STICH trial concluded that CABG increased the quality-adjusted life expectancy compared with MT alone at an increased cost ($63 989) although the latter was within the prespecified benchmark for good value within the US health care system ($100 000).^2^ The authors found that patients randomized to CABG+OMT gained 0.45 QALYs compared with OMT alone over a 10-year follow-up, whereas the REVIVED trial has identified no difference in QALYs between groups, albeit over a slightly shorter time horizon (median follow-up of 3.4 years). The difference in QALYs, in turn, is driven primarily by a differential treatment effect in relation to all-cause mortality. While it remains possible that this may relate to fundamental differences between CABG and PCI, direct study-level comparison of STICH and REVIVED is hampered by substantial differences between trial populations^17^ (as exemplified by the difference in age with patients enrolled in REVIVED being 10 years older than those in STICH) and major differences in use of device and MT in the 2 trials.

Economic analyses of revascularization by PCI have also been performed for the COURAGE (Clinical Outcomes Utiliz- ing Revascularization and Aggressive Drug Evalu- ation) and ORBITA (Objective Randomised Blinded Investigation with optimal medical Therapy of Angioplasty in stable angina) trials in patients with stable coronary artery disease although these studies specifically excluded patients with severe left ventricle systolic dysfunction and multivessel disease. Notwithstanding the important differences in target populations, different settings (United States and United Kingdom), modeling assumptions (within trial and model based), and comparators (best MT and placebo), the results of the aforementioned analyses support the view of some disparity in the economic value of PCI.^18,19^

A strength of this economic analysis was the relatively long study follow-up period (up to 8 years; median, 3.4 years). This offers a good understanding of the medium- to long-term costs and benefits of the strategies being evaluated. While a lifetime analysis could have been conducted, the results of this 8-year analysis indicated that this was unnecessary. Given the clinical, cost-related, and health-related quality-of-life results that we have reported, there seems to be unlikely a scenario in which long-term costs and outcomes would make PCI a cost-effective treatment, and we think that it is reasonable to conclude that estimated cost-effectiveness over an 8-year time horizon is generalizable to the patient’s lifetime. For this reason, no modeling to extrapolate evidence from the trial to the long term was considered necessary.

By using regression analyses in our study, we aimed to provide an unbiased estimate of the expected costs and health outcomes associated with each treatment, as should be the objectives of any cost-effectiveness analysis. By describing the relationship between the outcome of interest and treatment assignment, it helped us identify the expected costs and outcomes associated with the comparator treatment. The regression framework also enabled a characterization of the decision uncertainty from which an assessment of the need for any additional research could be made. A note that the uncertainty expressed in the cost-effectiveness estimates presented relate to second-order uncertainty (uncertainty around the mean for the average patient with severe ILVD) and is subject to structural uncertainty (uncertainty relating to the form of regression model implemented). On the latter, model specification may hinder an appropriate reflection of parameter uncertainty, which may be reflected in the relatively narrow CIs of the predicted total costs. With its advantages and disadvantages, alternative modeling has been implemented through a seemingly unrelated regression model.

The study has some limitations. The resource uses collected within the REVIVED trial are believed to be comprehensive though the aim of our study was not to estimate the total cost burden of ILVD but to capture cost and QALY differences between therapies here under scrutiny. Nevertheless, and as with many trial analyses, there are practical limits to what has been collected. For example, the total number of clinical tests performed on trial patients is likely to exceed what was captured in the case report form as only mandated tests to inform the specified clinical outcomes were captured. The total cost of managing these patients is likely to be appreciably higher than we have estimated; however, these uncaptured costs are likely to be distributed evenly between groups. Another potential limitation of our work is that medication data were collected only for the initial 2 years of follow-up. These limitations apply to both study arms and are unlikely to impact the overall conclusions of the analysis. A sensitivity analysis using alternative unit costs from NHS Reference Costs 2020/2021^20^ showed that the cost-effectiveness results were robust to changes to these costs.

Health-related quality of life collected via the EQ-5D-5L questionnaire contained a proportion of missing data. This was due to a small proportion of patients being lost to follow-up, being too unwell to complete the questionnaires, and limitations in face-to-face visits due to the COVID-19 pandemic. It was assumed that systematic differences between the missing and observed EQ-5D index scores existed, but these could be entirely explained by other observed variables. Thus, multiple imputation was used to address missingness, generate multiple imputed data sets, and achieve a similar time frame of analysis, providing a fuller understanding of the health-related quality-of-life data. This approach allowed us to account for any gaps or missing values in the per-patient QALY calculations and ensure a comprehensive evaluation of the health outcomes for both the OMT and PCI+OMT arms.

Finally, our analysis is based specifically on costs relating to the UK NHS and cannot, therefore, be directly transferable to other health systems. The costs of PCI are relatively low in the publicly funded NHS by comparison with privately funded health care systems, such as the United States. If similar health resource consumption and health-related quality of life are assumed, a higher cost for PCI would augment overall costs further and further increase its negative economic impact.

In summary, our results have identified that, for patients with severe ILVD in the United Kingdom, revascularization using PCI in addition to OMT is not considered to be cost-effective when compared with OMT alone, given its additional cost. These conclusions were robust to different modeling assumptions and unit costs. Routine use of PCI for the treatment of severe ILVD does not seem to be a justifiable use of the UK NHS resources.

## APPENDIX

REVIVED Sites and Investigators: Guy’s and St Thomas’ Hospital: Prof Divaka Perera (principal investigator); Prof Amedeo Chiribiri, Prof Gerry Carr-White, Dr Antonis Pavlidis, Prof Simon Redwood, Dr Brian Clapp, Prof Aldo Rinaldi, Dr Haseeb Rahman, Dr Natalia Briceno (coinvestigators); Sophie Arnold, Amy Raynsford, Karen Wilson, Lucy Clack (coordinators). Golden Jubilee National Hospital, Glasgow: Prof Mark Petrie (principal Investigator); Dr Margaret McEntegart, Dr Stuart Watkins, Dr Aadil Shaukat, Dr Paul Rocchiccioli (Coinvestigators); Marion McAdam, Elizabeth McPherson, Louise Cowan, Marie Wood (Coordinators). Barts Heart Centre, London: Dr Roshan Weerackody (principal investigator); Dr Ceri Davies, Dr Elliot Smith, Dr Bhavik Modi (coinvestigators); Bindu Mathew, Oliver Mitchelmore, Rita Adrego, Mervyn Andiapen (coordinators). Royal Bournemouth Hospital: Dr Peter O’Kane (principal investigator); Dr Jehangir Din (coinvestigator); Sarah Kennard, Sarah Orr, Cathie Purnell (coordinators). Leeds General Infirmary: Prof John Greenwood (principal investigator); Dr Jonathan Blaxill, Dr Abdul Mozid (coinvestigators); Michelle Anderson, Kathryn Somers (coordinators). Royal Victoria Hospital, Belfast: Dr Lana Dixon (principal investigator); Dr Simon Walsh, Dr Mark Spence (coinvestigators); Patricia Glover, Caroline Brown (coordinators). King’s College Hospital, London: Dr George Amin-Youssef (principal investigator); Prof Ajay Shah, Prof Theresa McDonagh, Dr Jonathan Byrne, Dr Nilesh Pareek (coinvestigators); Jonathan Breeze, Catherine Antao (coordinators). Bristol Royal Infirmary: Dr Kalpa De Silva (principal investigator); Dr Julian Strange, Dr Tom Johnson, Dr Angus Nightingale (coinvestigators); Laura Gallego, Cristina Medina (coordinators). Glenfield Hospital, Leicester: Prof Anthony Gershlick (principal investigator); Prof Gerald McCann, Dr Andrew Ladwiniec, Prof Iain Squire (coinvestigators); Joanna Davison, Kris Kenmuir-Hogg (coordinators). St George’s Hospital, London: Prof James Spratt (principal investigator); Dr Claudia Cosgrove, Dr Rupert Williams, Dr Sam Firoozi, Dr Pitt Lim (coinvestigators); Giovanna Bonato, Vennessa Sookhoo (coordinators). Pinderfields Hospital, Wakefield: Dr Dwayne Conway (principal investigator); Dr Paul Brooksby (coinvestigator); Judith Wright, Donna Exley (coordinators). New Cross Hospital, Wolverhampton: Dr James Cotton (principal investigator); Dr Richard Horton (coinvestigator); Stella Metherell, Andrew Smallwood (coordinators). Kettering General Hospital: Dr Kai Hogrefe (principal investigator); Dr Adrian Cheng (coinvestigator); Charmaine Beirnes, Sian Sidgwick (coordinators). Royal Free Hospital, London: Dr Tim Lockie (principal investigator); Dr Niket Patel, Dr Roby Rakhit (coinvestigators); Nina Davies, Angelique Smit (coordinators). Manchester Royal Infirmary: Dr Fozia Ahmed (principal investigator); Dr Cara Hendry, Dr Farzin Fath-Odoubadi, Dr Douglas Fraser, Dr Mamas Mamas (coinvestigators); Anu Oommen, Thabitha Charles (coordinators). Royal Infirmary of Edinburgh: Dr Miles Behan (principal investigator); Dr Alan Japp (coinvestigator); Belinda Rif (coordinator). Sunderland Royal Hospital: Dr Nicholas Jenkins (principal investigator); Dr Sam McClure (coinvestigator); Pauline Oates, Karen Martin (coordinators). Wythenshawe Hospital: Dr Eltigani Abdelaal (principal investigator); Dr Jaydeep Sarma, Dr Sanjay Shastri, Dr Jo Riley (coinvestigators); Sarra Giannopoulou, Sophie Quinn (coordinators). Liverpool Heart and Chest Hospital: Dr Pradeep Magapu (principal investigator); Prof Rod Stables, Dr David Wright (coinvestigators); Janet Barton, Nichola Clarkson (coordinators). Southampton General Hospital: Dr Michael Mahmoudi (principal investigator); Dr Andrew Flett, Prof Nick Curzen (coinvestigators); Judith Radmore, Sam Gough (coordinators). Royal Devon & Exeter Hospital: Dr Andrew Ludman (principal investigator); Dr Hibba Kurdi (coinvestigator); Samantha Keenan (coordinator). University Hospitals Coventry and Warwickshire: Prof Prithwish Banerjee (principal investigator); Dr Luke Tapp (coinvestigator); Nigel Edwards, Catherine Gibson (coordinators). Lister Hospital, Stevenage: Dr Neville Kukreja (principal investigator); Dr Mary Lynch (coinvestigator); Claire Barratt (coordinator). The James Cook University Hospital, Middlesbrough: Dr Mark de Belder (principal investigator); Dr Jeet Thambyrajah, Dr Neil Swanson (coinvestigators); Cath Richardson, Bev Atkinson (coordinators). Derriford Hospital, Plymouth: Dr Girish Viswanathan (principal investigator); Darren Waugh (coordinator). Worcestershire Acute Hospitals: Dr Helen Routledge (principal investigator); Dr Jasper Trevelyan (coinvestigator); Angela Doughty (coordinator). Worthing Hospital: Dr Nick Pegge (principal investigator); Dr Sukhbir Dhamrait (coinvestigator); Sally Moore (coordinator). Blackpool Victoria Hospital: Dr Gavin Galasko (principal investigator); Dr Christopher Cassidy (coinvestigator); Natalia Waddington (coordinator). Dorset County Hospital: Dr Tim Edwards (principal investigator); Dr Javed Iqbal, Dr Fraser Witherow (coinvestigators); Jenny Birch, Melanie Munro (coordinators). Salisbury District Hospital: Dr Tim Wells (principal investigator); Dr Manas Sinha (coinvestigator); Linda Frost (coordinator). Birmingham Heartlands Hospital: Dr Kaeng Lee (principal investigator); Dr James Beattie, Dr Mike Pitt (coinvestigators); Alan Chung (coordinator). Great Western Hospital, Swindon: Dr Steve Ramcharitar (principal investigator); Laura McCafferty (coordinator). Ninewells Hospital, Dundee: Dr Thomas Martin (principal investigator); Dr John Irving, Dr Zaid Iskandar (coinvestigators); Anita Hutcheon (coordinator). Northern General Hospital, Sheffield: Dr Julian Gunn (principal investigator); Dr Abdallah Al-Mohammad (coinvestigator); Michael Agyemang (coordinator). Queen Alexandra Hospital, Portsmouth: Dr Huw Griffiths (principal investigator); Prof Paul Kalra (coinvestigator); Serena Howe (coordinator). Royal Oldham Hospital: Dr Tim Gray (principal investigator); Dr Jolanta Sobolewska (coinvestigator); Louise Morby (coordinator). Basingstoke and North Hampshire Hospital: Dr Jason Glover (principal investigator); Dr James Beynon (coinvestigator); Janet Knight (coordinator). North Wales Cardiac Centre: Dr Paul Das (principal investigator); Dr Chris Bellamy (coinvestigator); Emily Harman (coordinator). The York Hospital: Mr Maurice Pye (principal investigator); Dr Simon Megarry (coinvestigator); Yvonne McGill, Heidi Redfearn (coordinators).

## ARTICLE INFORMATION

### Sources of Funding

This trial was sponsored by King’s College London and funded by the National Institute for Health and Care Research Health Technology Assessment Program (10/57/67). The arrhythmia analyses were supported by the British Heart Foundation (fellowship FS/CRTF/21/24190 and the King’s British Heart Foundation Center of Research Excellence grant RE/18/2/34213).

### Disclosures

None.

### Supplemental Material

Supplemental Methods

Tables S1–S10

References ^21–24^

## Supplementary Material


