# Supplementary material for: Percutaneous Revascularization for Ischemic Left Ventricular Dysfunction: Cost-Effectiveness Analysis of the REVIVED-BCIS2 Trial
Source: Circ Cardiovasc Qual Outcomes. 2023 Nov 6;17(1):e010533. doi: 10.1161/CIRCOUTCOMES.123.010533 (PMC10782932; doi:10.1161/CIRCOUTCOMES.123.010533)
Supplement: Supplementary file 1 [file hcq-17-e010533-s001.pdf]

SUPPLEMENTARY MATERIAL

Percutaneous Revascularisation for Ischemic Left Ventricular Dysfunction: Cost-Effectiveness analysis of the REVIVED-BCIS2 Trial

Contents

Supplemental Methods……………………………………………………………………………………………2

Missing data imputation……………………………………………………………………………………3

Supplemental Tables……………………………………………………………………………………………4

Table S1. Baseline clinical characteristics by treatment group………………………………………4

Table S2. Unit cost of resources used…………………………………………………………………5

Table S3. Imputed EQ5D index scores…………………………………………………………………7

Table S4. Average number of days of hospitalisation…………………………………………………8

Table S5. Number (%) of patients with new implantable devices by visit……………………………9

Table S6. Distribution of medications by visit and treatment group…………………………………10

Table S7. Proportion of individuals subject to clinical investigations by visit and treatment group……………………………………………………………………………………………………11

Table S8. Observed Total Cost and QALYs by treatment group………………………………………12

Table S9. Generalised Linear Models results…………………………………………………………13

Table S10. Results from the SUR model………………………………………………………………14

Missing Data Imputation

This technical note aims to provide a detailed explanation of the missing data imputation model utilised in the analysis. The imputation model is employed to handle missing values and ensure robustness of the results. The following sections describe the key aspects of the imputation model, including the assumptions made, the number of imputed datasets generated and the merging process of imputed results.

Imputation Model Details:

The missing data imputation model employed in this analysis follows a multiple imputation using chained equations (MICE) approach.[21] MICE is a widely used and flexible imputation method that addresses missing data by iteratively imputing each variable with missing values using regression models. The imputation process takes advantage of observed data patterns and relationships between variables to estimate the missing values. MICE incorporate uncertainty by generating multiple imputed datasets. In each iteration, the missing values are imputed based on the available data and the estimated regression models.[12] The imputation process is repeated several times, typically until convergence is achieved, resulting in M imputed datasets. Each imputed dataset represents a plausible set of values that accounts for the uncertainty associated with the missing data.

By generating multiple imputed datasets, MICE allow for capturing the variability introduced by imputing missing values.[22] This approach recognizes that there is inherent uncertainty in imputing missing data and provides a robust framework for conducting statistical analyses. After imputation, the M imputed datasets can be analysed separately using standard statistical methods, and the results can be combined using Rubin's rule to obtain valid and reliable estimates that account for the uncertainty introduced by the imputation process.

All the MICE models were estimated in STATA18 using the command ice.

Implementation in STATA:

The command ice is used to initiate the imputation process. The variable of interest with missing data, EQ5D score, is specified, along with several auxiliary variables including Hypertension, BMI, Ethnicity, and NYHA risk classification. These auxiliary variables are utilized in the imputation model to predict the missing values of EQ5D score (Table S3). It is important to note that the auxiliary variables should be complete without any missing values. The m option is set to 50, indicating that 50 imputed datasets will be created. The match option implies that the imputed datasets will be created using a matching algorithm.

In summary, the ice command performs multiple imputation using chained equations in Stata, incorporating auxiliary variables to predict missing values of the variable EQ5D score. The resulting imputed datasets with 50 imputations generated.

Assumptions:

The MICE (Multiple Imputation using Chained Equations) approach relies on several key assumptions when imputing missing data.[23] These assumptions play a crucial role in ensuring

the validity of the imputation process and the subsequent analyses conducted using the imputed data. There are some assumptions in MICE such as, Missingness Mechanism and Linearity and Correct Specification of Imputation Models. However, the main assumption of MICE is:

Missing at Random (MAR): This assumption states that the missingness of data can be explained by observed variables and is not dependent on unobserved variables after accounting for the observed ones. In other words, the probability of data being missing can be predicted by the available information.

Number of Imputed Datasets:

To capture the uncertainty associated with imputed values, a total of 50 imputed datasets were generated using the imputation model. Multiple imputed datasets allow for comprehensive analysis by accounting for the variability resulting from the imputation process.

Merging Imputed Results - Rubin's Rule:

After generating the imputed datasets, the imputed results were merged using Rubin's rule. Rubin's rule combines the estimates and variances from each imputed dataset, yielding valid and reliable inferences that account for the uncertainty introduced by the imputation model.[12]

Results of Imputation:

The imputed results provide a complete dataset, eliminating any missing values. This allows for a comprehensive analysis without the need to exclude cases due to missing data.

Supplemental Tables

Table S1. Baseline clinical characteristics by treatment group

	Percutaneous coronary intervention + optimal medical therapy (n=347)	***Optimal Medical Therapy (n=353)***
Baseline variables		
Age - yrs	70.0 ± 9.0	68.8 ± 9.1
BMI	28.4 ± 5.5	28.7 ± 5.4
Sex (Male) – no. (%)	302 (87)	312 (88)
Ethnicity – no. (%)		
Black	3 (1)	3 (1)
Asian	32 (9)	17 (5)
Caucasian	306 (88)	328 (93)
Mixed, other or not reported	6 (2)	5 (1)
NYHA class – no./total no. (%		
I or II	265/345 (77)	248/350 (71)
III or IV	80/345 (23)	102/350 (29)
Smoking – no. (%)		
Current/Ex	243 (70)	267 (76)
Never	104 (30)	86 (24)
BCIS Jeopardy score – median (IQR)	10 (8-12)	10 (8-12)
Hypertension – no./total no. (%)	184/347 (53)	207/352 (59)
Diabetes – no. (%)	136 (39)	153 (43)
Previous MI - no. (%)	175 (50)	197 (56)
Previous PCI – no. (%)	66 (19)	76 (22)
Previous CABG – no. (%)	12 (3)	22 (6)
EQ5D baseline – mean (SD)	0.67 ± 0.25	0.66 ± 0.27

Note: BMI= body mass index, NYHA= New York heart association, BCIS=British cardiovascular intervention society, MI= myocardial infarction, CABG= coronary artery bypass grafting, EQ-5D-5L = EuroQol 5-Dimension 5-Level

Table S2. Unit cost of resources used

Category	*Input*	*Unit cost*	*NHS main code*
Unplanned revascularization	PCI (short stay)	£3,413.00	EY40
	PCI (long stay)	£7,844.00	
	CABG (short stay)	£15,319.00	ED26
	CABG (long stay)	£19,383.00	
Planned revascularization	PCI	£6,675.00	EY40
Clinical investigations	Blood sampling	£1.20	DAPS0
	Haemoglobin	£1.20	
	Creatinine	£1.20	
	Lipids	£1.20	
	Cholesterol	£1.20	
	LDL	£1.20	
	HDL	£1.20	
	Triglyceride	£1.20	
	BNP	£7.40	
	NT-proBNP	£7.40	
	HbA1c	£1.20	
	Troponin	£1.20	
	Echocardiogram	£86.70	
*Implantable devices (base case)**	CRT	£9,616.69	-
	CRT-D	£20,727.97	DEV15
	ICD only	£17,707.44	DEV14
*Implantable devices (scenario analysis)**	CRT	£9,616.69	-
	CRT-D	£7,107.75	-
	ICD only	£7,986.93	-
Hospital visits	Day case	*£737.68*	*EB03*
Hospitalizations	Ward	£511.36	*EB03*
	Ventilation	£2,501.84	*XC0*
	Non-ventilation	£1,604.00	XC0

* Health system advice suggested that the ranking of the unit costs for implantable devices in the NHS Reference Costs 2021/22, with CRT (£9,617) being more expensive than CRT-D (£7,108), was not as expected.[7] Thus, devices were costed according to the methodology used to inform NICE's 2014 Technology Appraisal,

based on the NHS Reference Costs 2011/12 updated to 2021 prices, and estimated to be £17,707, £9,617 and £20,728 for ICD, CRT-P and CRT-D, respectively.[24]

Note: PCI = percutaneous coronary intervention, CABG= coronary artery bypass grafting, LDL= low-density lipoprotein, HDL = high-density lipoprotein, BNP = B-type natriuretic peptide, NT-proBNP = N-terminal pro B-type natriuretic peptide, HbA1c = hemoglobin A1c, CRT= cardiac resynchronization therapy, ICD = implantable cardioverter defibrillator, CRT-D = CRT with defibrillator.

Table S3: Imputed EQ5D index scores.

	Percutaneous coronary intervention + Optimal medical therapy (n = 347)			***Optimal medical therapy (n = 353)***		
Visit	***Mean***	***[95% CI]***		***Mean***	***[95% CI]***	
1Y	0.66	0.66	0.67	0.63	0.63	0.64
2Y	0.58	0.57	0.59	0.56	0.56	0.57
3Y	0.53	0.52	0.54	0.53	0.52	0.54
4Y	0.51	0.5	0.52	0.49	0.49	0.5
5Y	0.49	0.48	0.5	0.46	0.45	0.47
6Y	0.48	0.47	0.49	0.46	0.45	0.47
7Y	0.47	0.47	0.48	0.45	0.45	0.46
8Y	0.47	0.46	0.48	0.45	0.45	0.46

Note: *Y = years, CI = confidence interval.*

Table S4. Average number of days of hospitalisation

	Percutaneous coronary intervention + optimal medical therapy (n = 108)			Optimal medical therapy (n=103)		
Visit	***N***	***Mean***	***(95% CI)***	***N***	***Mean***	***(95% CI)***
6M	18	12.33	4.23 – 20.42	26	7.62	5.27 – 9.95
1Y	29	10.72	6.12 – 15.31	13	9.92	4.45 – 15.38
2Y	24	15.63	9.46 – 21.78	20	14.35	9.57 – 19.12
3Y	13	14.08	7.50 – 20.65	14	14.07	6.88 – 21.25
4Y	13	8.31	1.68 – 14.92	12	33.58	11.59 – 78.76
5Y	4	12	1.63 – 25.63	11	21.55	5.52 – 37.56
6Y	4	11.75	4.18 – 19.31	6	22.33	1.59 – 43.06
7Y	3	9.83	2.63 – 17.03	1	1	.
Overall*	108	12.25	9.66 - 14.84	103	15.39	9.51 - 21.28

M= months, Y= years, N= number, CI= confidence interval

** T-test was performed. Difference is not statistically significant, p-value = 0.2932.*

Table S5. Number (%) of patients with new implantable devices by visit

	Percutaneous coronary intervention + optimal medical therapy (n=347)				*Optimal Medical Therapy (n=353)*			
Visit	*ICD*	*CRT-D*	*CRT-P*	*Total*	*ICD*	*CRT-D*	*CRT-P*	*Total*
Before BL*	46	31	5	82	33	38	5	76
(%)	52.4	41.5	6.1	100	35.5	57.9	6.6	100
0 to <6 months	29	25	1	55	43	26	1	70
(%)	52.7	45.5	1.8	100	61.4	37.1	1.4	100
6 to <12 months	9	5	0	14	19	2	1	22
(%)	64.3	35.7	0	100	86.4	9.1	4.6	100
1 to <2 years	6	6	0	12	13	7	1	21
(%)	50	50	0	100	61.9	33.3	4.8	100
2 to <3 years	5	0	2	7	4	1	1	6
(%)	71.4	0	28.6	100	66.7	16.7	16.7	100
3 to <4 years	0	0	2	2	0	0	0	0
(%)	0	0	100	100	0	0	0	0
4 to <5 years	2	0	0	2	0	0	0	0
(%)	100	0	0	100	0	0	0	0
5 to <6 years	0	1	0	1	1	0	0	1
(%)	0	100	0	100	100	0	0	100
Overall (post-baseline)†	51	37	5	93	80	36	4	120
(%)	54.8	39.8	5.4	100	66.7	30	3.3	100

* Before baseline data on devices shown for completeness, however not included in the economic analysis.

† Chi squared test was performed. Difference is not statistically significant, p-value =0.205.
Note: 121 devices were implanted after baseline in the OMT group; one patient was missing the date of implantation, hence we present data on n=120 in the OMT group.

BL= baseline, ICD = implantable cardioverter defibrillator, CRT-D = cardiac resynchronization therapy with defibrillator, CRT-P = cardiac resynchronization therapy pacemaker

Table S6. Distribution of medications by visit and treatment group

Medication	Baseline				6 Month				1 year				2 year			
	PCI+OMT		OMT alone		PCI+OMT		OMT alone		PCI+OMT		OMT alone		PCI+OMT		OMT alone	
	N	Prop	N	Prop	N	Prop	N	Prop	N	Prop	N	Prop	N	Prop	N	Prop
Aspirin	347	69%	351	71%	323	76%	324	68%	311	68%	314	68%	287	62%	282	62%
Antiplatelet	347	40%	352	39%	323	84%	324	36%	310	78%	314	28%	287	41%	282	28%
beta blocker	347	91%	353	90%	323	93%	324	94%	311	95%	314	92%	287	92%	282	94%
Warfarin	345	14%	353	16%	323	11%	323	15%	311	12%	314	14%	286	9%	283	12%
Other anticoagulant	297	22%	302	19%	299	24%	296	23%	302	24%	300	21%	284	26%	282	24%
ACE inhibitor	345	69%	352	67%	323	61%	324	57%	311	58%	314	53%	285	50%	282	44%
Angiotensin blocker	342	16%	351	17%	321	17%	324	19%	311	18%	313	21%	284	20%	281	18%
Aldosterone	346	51%	351	48%	323	49%	323	57%	311	54%	313	55%	286	54%	281	57%
Statin	347	85%	353	86%	323	85%	324	88%	311	89%	313	89%	286	88%	282	85%
Amiodarone	346	5%	353	4%	323	5%	324	5%	311	6%	314	6%	285	6%	282	7%
Digoxin	346	6%	353	9%	323	7%	324	8%	311	7%	313	6%	285	8%	282	7%
Loop or Thiazide	346	66%	351	66%	323	67%	324	67%	311	63%	314	62%	286	60%	282	63%
Insulin	345	11%	352	13%	323	10%	323	11%	311	12%	314	11%	285	11%	283	14%
Oral Hypoglycaemic	345	29%	353	30%	323	28%	323	29%	311	29%	314	27%	285	28%	282	29%
Entresto	93	16%	88	26%	106	25%	112	33%	132	30%	133	33%	174	32%	163	37%

Note : PCI = percutaneous coronary intervention, OMT = optimal medical therapy, N = number, prop = proportion

Table S7. Proportion of individuals subject to clinical investigations by visit and treatment group

Clinical investigations	Percutaneous coronary intervention + Optimal medical therapy								Optimal medical therapy							
	Baseline		6M		1Y		2Y		Baseline		6M		1Y		2Y	
	Mean	SD	Mean	SD	Mean	SD	Mean	SD	Mean	SD	Mean	SD	Mean	SD	Mean	SD
Haemoglobin	0.99	0.1	.	.	.	.	.	.	0.98	0.15	.	.	.	.	.	.
Creatinine	0.99	0.1	.	.	.	.	.	.	0.99	0.12	.	.	.	.	.	.
Total cholesterol	0.88	0.3	.	.	.	.	.	.	0.9	0.3	.	.	.	.	.	.
LDL	0.68	0.5	.	.	.	.	.	.	0.69	0.46	.	.	.	.	.	.
HDL	0.79	0.4	.	.	.	.	.	.	0.81	0.39	.	.	.	.	.	.
Triglyceride	0.84	0.4	.	.	.	.	.	.	0.85	0.36	.	.	.	.	.	.
BNP	0.07	0.3	0.21	0.4	0.1	0.3	0.07	0.25	0.07	0.25	0.18	0.39	0.07	0.26	0.08	0.27
NT-proBNP	0.84	0.4	0.73	0.5	0.65	0.5	0.54	0.5	0.81	0.39	0.71	0.45	0.61	0.49	0.54	0.5
HbA1C	0.27	0.5	.	.	.	.	.	.	0.25	0.43	.	.	.	.	.	.
Troponin T	0	0	0.15	0.4	0.13	0.3	.	.	0.05	0.21	0.2	0.4	0.1	0.3	.	.
Troponin I	0	0	0.33	0.5	0.25	0.4	.	.	0.13	0.34	0.32	0.47	0.2	0.4	.	.
Echo	.	.	0.87	0.3	0.82	0.4	.	.	.	.	0.85	0.36	0.81	0.39	.	.
HbA1c mol	0.81	0.4	.	.	.	.	.	.	0.79	0.41	.	.	.	.	.	.

Note: M = months, Y = years, SD = standard deviation, LDL= low-density lipoprotein, HDL = high-density lipoprotein, BNP = B-type natriuretic peptide, NT-proBNP = N-terminal pro B-type natriuretic peptide, HbA1c = hemoglobin A1c

Table S8. Observed Total Cost and QALYs by treatment group

Group	*Obs*	*Mean*	*[95% CI]*
QALYs			
Percutaneous coronary intervention + Optimal medical therapy	347	4.197	3.925 – 4.468
Optimal medical therapy	353	4.145	3.881 – 4.408
Incremental QALYs		0.052	-0.326 – 0.430
Costs			
Percutaneous coronary intervention + Optimal medical therapy	347	£21,674	£19,722 - £23,626
Optimal medical therapy	353	£15,882	£13,958 - £17,806
Incremental Cost		£5,791	£3,056 - £8,528

Note: Observed incremental cost-effectiveness ratio = £111,365/QALY gained (north east quadrant of cost effectiveness plane)

QALYs: quality adjusted life years, Obs = number of observations CI = confidence interval

Table S9. Generalised Linear Models results

Family:	***Gamma distribution***					
Outcome:	***Log (Total cost)***			***Log (Total QALYs)***		
Variables	***Coef.***	***p-value***	***Std. err.***	***Coef***	***p-value***	***Std. err.***
Treatment group	0.362	0.000	0.079	-0.016	0.878	0.101
Age centered	0.005	0.270	0.005	-0.032	0.000	0.006
BMI	0.026	0.001	0.008	-0.001	0.926	0.010
Sex (female)	-0.244	0.039	0.118	0.200	0.230	0.166
Ethnicity (ref. =Caucasian)						
Afro-Caribbean	-0.663	0.113	0.418	-0.443	0.519	0.684
Asian	-0.155	0.341	0.163	-0.052	0.804	0.207
Other	-0.048	0.855	0.264	0.181	0.569	0.318
NYHA scale (ref. = I)						
II	0.034	0.742	0.104	-0.473	0.000	0.121
III	0.139	0.254	0.122	-1.705	0.000	0.184
IV	0.366	0.274	0.334	-2.618	0.000	0.438
Smoking (ref. = Never)						
Current	-0.034	0.777	0.120	-0.402	0.013	0.157
Ex	0.013	0.889	0.092	-0.183	0.082	0.105
BCIS Jeopardy (ref. = Mild)						
Moderate	0.378	0.127	0.248	-0.382	0.351	0.405
Severe	0.360	0.143	0.246	-0.603	0.126	0.402
Hypertension	0.045	0.577	0.081	-0.224	0.011	0.087
Diabetes	0.058	0.471	0.081	-0.442	0.000	0.114
Previous MI	-0.030	0.722	0.085	0.096	0.395	0.112
Previous PCI	0.186	0.076	0.104	-0.158	0.222	0.129
Previous CABG	-0.081	0.664	0.186	-0.173	0.505	0.258
Hospital admission	0.136	0.108	0.085	-0.487	0.000	0.102
EQ5D baseline	-----		-----	2.518	0.000	0.250

Note: The BCIS jeopardy score is a simple method for estimating the amount of myocardium at risk on the basis of the particular location of coronary artery stenoses. The maximum score is 12 and a score ≥6 was required to be eligible for REVIVED. A score of 12 indicates proximal disease in all three major epicardial coronary arteries.

Coef = coefficient, Std err. = standard error, ref = reference, BMI= body mass index, NYHA= New York heart association, BCIS=British cardiovascular intervention society, MI= myocardial infarction, PCI = percutaneous coronary intervention, CABG= coronary artery bypass grafting, EQ-5D-5L = EuroQol 5-Dimension 5-Level

Table S10. Results from the SUR model

**Dependent:**	**Total Cost**			**Total QALY**		
Independent:	***Coef***	***Std. err.***	***P>t***	***Coef***	***Std. err.***	***P>t***
Treatment group	6379.50	492.22	0.00	-0.02	0.10	0.88
Age centered	95.02	28.40	0.00	-0.03	0.01	0.00
BMI	492.46	47.96	0.00	0.01	0.01	0.65
Sex	-3639.26	748.60	0.00	0.17	0.15	0.25
Ethnicity						
Afro-Caribbean	-9081.47	2653.77	0.00	-0.14	0.61	0.82
Asian	-2932.65	1024.31	0.00	0.09	0.25	0.71
Other	1186.42	1535.26	0.44	0.32	0.36	0.38
NYHA scale						
II	1185.86	663.82	0.07	-0.06	0.06	0.34
III	2214.01	778.34	0.00	-0.59	0.13	0.00
IV	6111.81	2123.36	0.00	-1.75	0.15	0.00
Smoking	159.02	286.90	0.58	-2.54	0.37	0.00
BCIS Jeopardy score	-48.14	100.28	0.63	-0.06	0.02	0.00
Hypertension	464.34	512.23	0.37	-0.26	0.10	0.02
Diabetes	372.66	511.07	0.47	-0.42	0.10	0.00
Previous MI	-683.23	527.55	0.20	0.02	0.10	0.85
Previous PCI	2821.00	650.48	0.00	-0.14	0.14	0.32
Previous CABG	-1586.75	1163.11	0.17	-0.08	0.22	0.71
Hospital admission	1841.20	527.44	0.00	-0.52	0.10	0.00
EQ5D baseline				2.39	0.20	0.00
Constant	-1229.51	1703.57	0.47	4.36	0.45	0.00

Note: The BCIS jeopardy score is a simple method for estimating the amount of myocardium at risk on the basis of the particular location of coronary artery stenoses. The maximum score is 12 and a score ≥6 was required to be eligible for REVIVED. A score of 12 indicates proximal disease in all three major epicardial coronary arteries. *Coef = coefficient, Std err. = standard error, BMI= body mass index, NYHA= New York heart association, BCIS=British cardiovascular intervention society, MI= myocardial infarction, PCI = percutaneous coronary intervention, CABG= coronary artery bypass grafting, EQ-5D-5L = EuroQol 5-Dimension 5-Level.*
