# Supplementary material for: Percutaneous Revascularization for Ischemic Left Ventricular Dysfunction: Cost-Effectiveness Analysis of the REVIVED-BCIS2 Trial
Source: Circ Cardiovasc Qual Outcomes. 2023 Nov 6;17(1):e010533. doi: 10.1161/CIRCOUTCOMES.123.010533 (PMC10782932; doi:10.1161/CIRCOUTCOMES.123.010533)
Supplement: Supplementary file 2 [file hcq-17-e010533-s002.docx]

Very brief summary

REVIVED-BCIS2 health economic analysis: PCI provides similar QOL benefits but at considerably higher cost compared to OMT alone

Twitter handles

@divaka_perera

@UKheartresearch

@hollymorgs
